# Poly[(μ_2_-nitrato-κ^2^ *O*:*O*′)(μ_2_-pyrimidin­ium-2-carboxyl­ato-κ^2^ *O*:*O*′)lithium(I)]

**DOI:** 10.1107/S1600536811019520

**Published:** 2011-05-28

**Authors:** Wojciech Starosta, Janusz Leciejewicz

**Affiliations:** a Institute of Nuclear Chemistry and Technology, ul.Dorodna 16, 03-195 Warszawa, Poland

## Abstract

In the structure of the title compound, [Li(C_5_H_4_N_2_O_2_)(NO_3_)]_*n*_, the Li^I^ ion is coordinated by two carboxyl­ate O atoms donated by two ligands and two nitrate O atoms in a distorted tetrahedral geometry. Li^I^ ions, bridged by carboxyl­ate O atoms, form mol­ecular ribbons composed of dimeric units. Two nitrate O atoms link the ribbons into mol­ecular layers parallel to (001). Hydrogen bonds are active between protonated heterocyclic N atoms as donors and carboxyl­ate O atoms as acceptors. The layers are held together by van der Waals inter­actions.

## Related literature

For the polymeric structures of some metal complexes with a pyrimidine-2-carboxyl­ate ligand, see: Rodríguez-Diéguez *et al.* (2007 ▶, 2008 ▶); Zhao & Liu (2010 ▶); Zhang *et al.* (2008*a* ▶). For structures built of monomeric mol­ecules, see: Kokunov & Gorbunova (2007 ▶); Antolić *et al.* (2000 ▶); Zhang *et al.* (2008*b* ▶); Suares-Varela *et al.* (2008 ▶). 
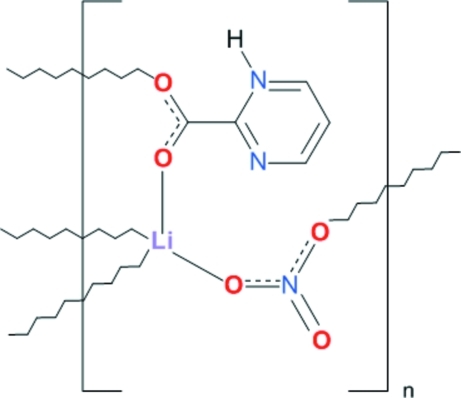


## Experimental

### 

#### Crystal data

[Li(C_5_H_4_N_2_O_2_)(NO_3_)]
*M* *_r_* = 193.05
Orthorhombic, 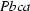

*a* = 12.403 (3) Å
*b* = 9.3290 (19) Å
*c* = 12.810 (3) Å
*V* = 1482.2 (5) Å^3^
*Z* = 8
Mo *K*α radiation
μ = 0.15 mm^−1^
*T* = 293 K
0.49 × 0.48 × 0.14 mm

#### Data collection

Kuma KM-4 four-circle diffractometer
Absorption correction: analytical (*CrysAlis RED*; Oxford Diffraction,2008 ▶) *T*_min_ = 0.782, *T*_max_ = 0.939
4248 measured reflections
2179 independent reflections
1504 reflections with *I* > 2σ(*I*)
*R*_int_ = 0.158
3 standard reflections every 200 reflections  intensity decay: 3.8%

#### Refinement

*R*[*F* ^2^ > 2σ(*F* ^2^)] = 0.050
*wR*(*F* ^2^) = 0.153
*S* = 0.97
2179 reflections
131 parameters
H atoms treated by a mixture of independent and constrained refinement
Δρ_max_ = 0.43 e Å^−3^
Δρ_min_ = −0.32 e Å^−3^

### 

Data collection: *KM-4 Software* (Kuma, 1996 ▶); cell refinement: *KM-4 Software*; data reduction: *DATAPROC* (Kuma, 2001 ▶); program(s) used to solve structure: *SHELXS97* (Sheldrick, 2008 ▶); program(s) used to refine structure: *SHELXL97* (Sheldrick, 2008 ▶); molecular graphics: *SHELXTL* (Sheldrick, 2008 ▶); software used to prepare material for publication: *SHELXTL*.

## Supplementary Material

Crystal structure: contains datablocks I, global. DOI: 10.1107/S1600536811019520/kp2322sup1.cif

Structure factors: contains datablocks I. DOI: 10.1107/S1600536811019520/kp2322Isup2.hkl

Additional supplementary materials:  crystallographic information; 3D view; checkCIF report

## Figures and Tables

**Table 1:** Selected bond lengths (Å)

O1—Li1	1.978 (3)
O11—Li1	1.967 (3)
Li1—O12^i^	2.001 (4)
Li1—O2^ii^	2.019 (3)

Symmetry codes: (i) 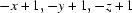; (ii) 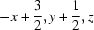.

**Table 2:** Hydrogen-bond geometry (Å, °)

*D*—H⋯*A*	*D*—H	H⋯*A*	*D*⋯*A*	*D*—H⋯*A*
N2—H2⋯O1^iii^	0.90 (3)	1.68 (3)	2.5762 (17)	174 (3)

Symmetry code: (iii) 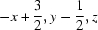.

**Fig. 1.**
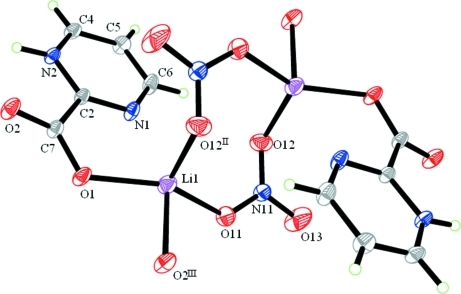
A fragment of the structure with the atom labeling scheme. Non-hydrogen atoms are shown as 50% elipsoids. Symmetry code: (I) -x + 3/2, y - 1/2, z; (II) -x + 1, -y + 1, -z + 1; (III) -x + 3/2, y + 1/2, z.

**Fig. 2.**
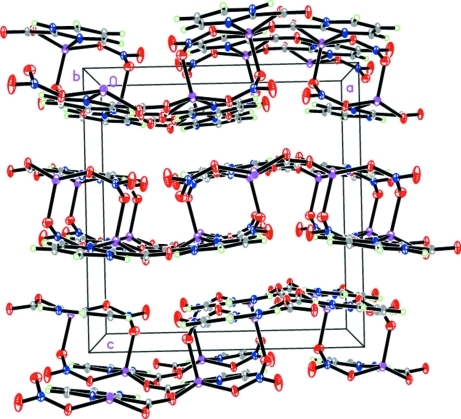
Packing of molecular layers viewed along the a axis.

## supplementary crystallographic information

### Comment

The structure of the title compound contains Li^I^ ions, each coordinated by two ligand carboxylato and two nitrato O atoms at the apieces of a distorded trigonal pyramid. Its base is composed of coplanar carboxylato O1, nitrato O11 and O12^II^ atoms (Fig. 1). The Li^I^ ion is shifted by 0.0548 (2)Å above this plane. The carboxylato O2^III^ atom is at the apex of the pyramid. The Li—O bond distances fall in the range from 1.967 (3) to 2.019 (3) Å, commonly observed in the structures of Li complexes with carboxylate ligands (Table 1). The Li1—N1 bond distance of 2.467 (3)Å as too long was not allowed for in coordination of the Li ion The pyrimidine ring is planar with r.m.s. of 0.0074 (2) Å]. A hydrogen atom attached to the hetero-ring N2 atom, clearly visible on the Fourier map, maintains the charge balance. It links the N2 atom with the carboxylato O^I^ atom *via* a hydrogen bond of 2.5762 (17) Å. Bond distances and bond angles within the pyrimidine ring do not differ from those reported earlier in the structures of other metal complexes with the title ligand. The carboxylate group C7/O1/O2 makes with the ring a dihedral angle of 14.81 (2)°. Two Li^I^ ions, one coordinated by the carboxylato O1 atom, the other by the second carboxylato O2 atom of the same ligand form molecular ribbons composed of dimeric units (Fig. 2). The latter bridged by nitrato O11 and O12^II^ atoms give rise to molecular layers. While the nitrato O11 atom coordinates a Li^I^ ion in one ribbon plane, the O12 atom is bonded along the crystal *c* axis to a Li^I^ ion in an adjacent ribbon; the O11—N11—O12 bond angle is 120.44 (17)°. The third nitrato O13 atomis not involved in the coordination. The NO_3_ group is planar with r.m.s. of 0.0023 (0) Å. It makes a dihedral angle of 28.8 (2)° with the ribbon plane. Since the bridging nitrate O12 atom is in a terminal position and it is bonded to the Li^I^ ion in the middle of an adjacent ribbon, a layer is formed. A sequence of open channels which propagate along crystal *a* direction form a layer parallel to the *ab* plane. The layers stacked along the crystal *c* direction are held together by van der Waals type interactions. A variety of polymeric molecular patterns have been recently observed in the structures of a number of divalent metal complexes with the title ligand, for example: Mn(II) (Rodríguez-Diéguez *et al.*, 2008; Zhang *et al.*, 2008*a*); Fe(II) and Co(II) (Rodríguez-Diéguez *et al.*, 2007; Zhao & Liu, 2010); Ca(II) Zhang *et al.*, 2008*a*), complexes. Structures built of monmeric molecules have been also reported: in a Ag(I) complex by Kokunov & Gorbunova, (2007); in a Cu(II) complex by Suares-Varela *et al.*, (2008) and Zhang *et al.*, (2008*a*). The structures of two Co(II) complexes have been determined by Antolić *et al.*, (2000) and Zhang *et al.*, (2008*b*).

### Experimental

50 mL of an aqueous solution containing 1 mmol of pyrimidine-2-carbonitrile (Aldrich) and 1 mmol of lithium nitrate hydrate were boiled with constant stirring under reflux for 6 h. After cooling to room temperature 1 N HNO_3_ was added dropwise until the pH reached 6. Then the solution was stirred for 3 h. After evaporation to dryness the residue was repeatedly dissolved in water and evaporated at room temperature until colourless single crystals of the title compound were deposited. The crystals were washed with cold methanol and dried in the air.

### Refinement

H atoms attached to pyridimiine-ring C atoms were placed at calculated positions with C—H=0.93 Å and treated as riding on the parent atoms with *U*_iso_(H)=1.2*U*_eq_(C). The H atom attached to pyrimidine ring N2 atom has been found from the Fourier map and refined isotropically.

### Crystal data

**Table:**

[Li(C_5_H_4_N_2_O_2_)(NO_3_)]	*F*(000) = 784
*M**_r_* = 193.05	*D*_x_ = 1.730 Mg m^−^^3^
Orthorhombic, *P**b**c**a*	Mo *K*α radiation, λ = 0.71073 Å
Hall symbol: -P 2ac 2ab	Cell parameters from 25 reflections
*a* = 12.403 (3) Å	θ = 6–15°
*b* = 9.3290 (19) Å	µ = 0.15 mm^−^^1^
*c* = 12.810 (3) Å	*T* = 293 K
*V* = 1482.2 (5) Å^3^	Plates, colorless
*Z* = 8	0.49 × 0.48 × 0.14 mm

### Data collection

**Table:**

Kuma KM-4 four-circle diffractometer	1504 reflections with *I* > 2σ(*I*)
Radiation source: fine-focus sealed tube	*R*_int_ = 0.158
graphite	θ_max_ = 30.1°, θ_min_ = 3.2°
profile data from ω/2θ scans	*h* = 0→17
Absorption correction: analytical (*CrysAlis RED*; Oxford Diffraction,2008)	*k* = 0→13
*T*_min_ = 0.782, *T*_max_ = 0.939	*l* = −18→18
4248 measured reflections	3 standard reflections every 200 reflections
2179 independent reflections	intensity decay: 3.8%

### Refinement

**Table:**

Refinement on *F*^2^	Primary atom site location: structure-invariant direct methods
Least-squares matrix: full	Secondary atom site location: difference Fourier map
*R*[*F*^2^ > 2σ(*F*^2^)] = 0.050	Hydrogen site location: inferred from neighbouring sites
*wR*(*F*^2^) = 0.153	H atoms treated by a mixture of independent and constrained refinement
*S* = 0.97	*w* = 1/[σ^2^(*F*_o_^2^) + (0.0702*P*)^2^ + 0.2975*P*] where *P* = (*F*_o_^2^ + 2*F*_c_^2^)/3
2179 reflections	(Δ/σ)_max_ < 0.001
131 parameters	Δρ_max_ = 0.43 e Å^−^^3^
0 restraints	Δρ_min_ = −0.32 e Å^−^^3^

### Special details

**Table:**

Geometry. All e.s.d.'s (except the e.s.d. in the dihedral angle between two l.s. planes) are estimated using the full covariance matrix. The cell e.s.d.'s are taken into account individually in the estimation of e.s.d.'s in distances, angles and torsion angles; correlations between e.s.d.'s in cell parameters are only used when they are defined by crystal symmetry. An approximate (isotropic) treatment of cell e.s.d.'s is used for estimating e.s.d.'s involving l.s. planes.
Refinement. Refinement of *F*^2^ against ALL reflections. The weighted *R*-factor *wR* and goodness of fit *S* are based on *F*^2^, conventional *R*-factors *R* are based on *F*, with *F* set to zero for negative *F*^2^. The threshold expression of *F*^2^ > σ(*F*^2^) is used only for calculating *R*-factors(gt) *etc*. and is not relevant to the choice of reflections for refinement. *R*-factors based on *F*^2^ are statistically about twice as large as those based on *F*, and *R*- factors based on ALL data will be even larger.

### Fractional atomic coordinates and isotropic or equivalent isotropic displacement parameters (Å^2^)

**Table:**

	*x*	*y*	*z*	*U*_iso_*/*U*_eq_	
N2	0.61057 (10)	0.04538 (11)	0.35126 (12)	0.0250 (3)	
O1	0.71796 (9)	0.39321 (10)	0.32671 (12)	0.0323 (3)	
O11	0.47373 (9)	0.63026 (13)	0.36709 (13)	0.0400 (4)	
N1	0.52999 (10)	0.27071 (12)	0.37133 (13)	0.0294 (3)	
C2	0.61507 (11)	0.18883 (13)	0.35586 (13)	0.0236 (3)	
N11	0.38359 (10)	0.62115 (13)	0.40961 (13)	0.0300 (3)	
C4	0.51530 (13)	−0.02147 (14)	0.35905 (15)	0.0290 (4)	
H4	0.5115	−0.1208	0.3538	0.035*	
O2	0.80624 (9)	0.18762 (11)	0.35001 (13)	0.0378 (4)	
C7	0.72441 (11)	0.26027 (14)	0.34338 (14)	0.0258 (3)	
C5	0.42357 (12)	0.05728 (16)	0.37473 (16)	0.0332 (4)	
H5	0.3565	0.0135	0.3806	0.040*	
C6	0.43471 (12)	0.20546 (16)	0.38159 (17)	0.0350 (4)	
H6	0.3737	0.2610	0.3937	0.042*	
O12	0.35866 (13)	0.51255 (13)	0.45999 (13)	0.0464 (4)	
O13	0.32128 (12)	0.72177 (17)	0.4017 (2)	0.0755 (7)	
Li1	0.6045 (2)	0.5149 (3)	0.3893 (3)	0.0362 (7)	
H2	0.671 (2)	−0.005 (3)	0.339 (2)	0.044 (6)*	

### Atomic displacement parameters (Å^2^)

**Table:**

	*U*^11^	*U*^22^	*U*^33^	*U*^12^	*U*^13^	*U*^23^
N2	0.0196 (5)	0.0171 (5)	0.0382 (8)	−0.0002 (4)	−0.0020 (5)	−0.0003 (5)
O1	0.0227 (5)	0.0175 (4)	0.0567 (8)	−0.0023 (3)	0.0012 (5)	0.0000 (4)
O11	0.0233 (6)	0.0398 (6)	0.0568 (10)	0.0039 (4)	0.0078 (5)	0.0067 (6)
N1	0.0190 (6)	0.0212 (5)	0.0479 (9)	0.0021 (4)	−0.0008 (5)	−0.0048 (5)
C2	0.0200 (6)	0.0184 (5)	0.0323 (8)	−0.0005 (4)	−0.0018 (6)	−0.0018 (5)
N11	0.0230 (6)	0.0287 (6)	0.0384 (8)	−0.0003 (4)	−0.0009 (6)	0.0007 (5)
C4	0.0260 (7)	0.0200 (5)	0.0410 (10)	−0.0042 (5)	−0.0013 (7)	0.0014 (5)
O2	0.0183 (5)	0.0241 (5)	0.0710 (10)	0.0023 (4)	0.0002 (6)	0.0032 (5)
C7	0.0199 (6)	0.0186 (5)	0.0388 (8)	−0.0011 (4)	−0.0017 (6)	−0.0029 (5)
C5	0.0193 (6)	0.0304 (6)	0.0499 (11)	−0.0065 (5)	−0.0018 (7)	0.0000 (7)
C6	0.0191 (6)	0.0308 (7)	0.0551 (12)	0.0021 (5)	0.0020 (7)	−0.0064 (7)
O12	0.0557 (8)	0.0362 (6)	0.0474 (9)	−0.0142 (6)	0.0041 (8)	0.0043 (6)
O13	0.0321 (7)	0.0554 (8)	0.139 (2)	0.0210 (6)	0.0136 (10)	0.0267 (11)
Li1	0.0294 (13)	0.0305 (11)	0.0486 (19)	0.0042 (10)	0.0029 (13)	0.0051 (12)

### Geometric parameters (Å, °)

**Table:**

N2—C4	1.3399 (19)	N11—O12	1.2404 (18)
N2—C2	1.3406 (16)	C4—C5	1.369 (2)
N2—H2	0.90 (3)	C4—H4	0.9300
O1—C7	1.2610 (16)	O2—C7	1.2234 (17)
O1—Li1	1.978 (3)	O2—Li1^i^	2.019 (3)
O11—N11	1.2466 (18)	C5—C6	1.392 (2)
O11—Li1	1.967 (3)	C5—H5	0.9300
N1—C2	1.3178 (18)	C6—H6	0.9300
N1—C6	1.3357 (19)	O12—Li1^ii^	2.001 (4)
N1—Li1	2.469 (3)	Li1—O12^ii^	2.001 (4)
C2—C7	1.5194 (19)	Li1—O2^iii^	2.019 (3)
N11—O13	1.2201 (18)		
			
C4—N2—C2	119.87 (13)	O2—C7—C2	119.35 (12)
C4—N2—H2	120.5 (15)	O1—C7—C2	113.12 (11)
C2—N2—H2	119.5 (15)	C4—C5—C6	117.36 (14)
C7—O1—Li1	122.76 (14)	C4—C5—H5	121.3
N11—O11—Li1	129.73 (15)	C6—C5—H5	121.3
C2—N1—C6	117.33 (11)	N1—C6—C5	122.29 (14)
C2—N1—Li1	104.42 (11)	N1—C6—H6	118.9
C6—N1—Li1	137.95 (11)	C5—C6—H6	118.9
N1—C2—N2	123.50 (13)	N11—O12—Li1^ii^	123.35 (15)
N1—C2—C7	118.44 (11)	O11—Li1—O1	147.4 (2)
N2—C2—C7	118.06 (12)	O11—Li1—O12^ii^	113.43 (18)
O13—N11—O12	120.90 (16)	O1—Li1—O12^ii^	98.94 (15)
O13—N11—O11	118.66 (15)	O11—Li1—O2^iii^	88.82 (12)
O12—N11—O11	120.44 (14)	O1—Li1—O2^iii^	88.11 (14)
N2—C4—C5	119.61 (12)	O12^ii^—Li1—O2^iii^	102.57 (16)
N2—C4—H4	120.2	O11—Li1—N1	100.51 (13)
C5—C4—H4	120.2	O1—Li1—N1	72.49 (10)
C7—O2—Li1^i^	156.13 (14)	O12^ii^—Li1—N1	93.28 (13)
O2—C7—O1	127.53 (13)	O2^iii^—Li1—N1	156.71 (19)

Symmetry codes: (i) −*x*+3/2, *y*−1/2, *z*; (ii) −*x*+1, −*y*+1, −*z*+1; (iii) −*x*+3/2, *y*+1/2, *z*.

### Hydrogen-bond geometry (Å, °)

**Table:**

*D*—H···*A*	*D*—H	H···*A*	*D*···*A*	*D*—H···*A*
N2—H2···O1^i^	0.90 (3)	1.68 (3)	2.5762 (17)	174 (3)

Symmetry codes: (i) −*x*+3/2, *y*−1/2, *z*.
